# An Open Label Non-inferiority Trial Assessing Vibriocidal Response of a Killed Bivalent Oral Cholera Vaccine Regimen following a Five Year Interval in Kolkata, India

**DOI:** 10.1371/journal.pntd.0003809

**Published:** 2015-05-29

**Authors:** Suman Kanungo, Sachin N. Desai, Jayanta Saha, Ranjan Kumar Nandy, Anuradha Sinha, Deok Ryun Kim, Barnali Bannerjee, Byomkesh Manna, Jae Seung Yang, Mohammad Ali, Dipika Sur, Thomas F. Wierzba

**Affiliations:** 1 National Institute of Cholera and Enteric Diseases, Kolkata, India; 2 International Vaccine Institute, Seoul, Republic of Korea; 3 Johns Hopkins Bloomberg School of Public Health, Baltimore, Maryland, United States of America; 4 PATH India Office, New Delhi, India; Massachusetts General Hospital, UNITED STATES

## Abstract

**Background:**

The bivalent killed oral cholera vaccine (OCV) provides 65% cumulative protection over five years. It remains unknown whether a boosting regimen can maintain protection in previously immunized populations. This study examines the immunogenicity and safety of an OCV regimen given five years following initial dosing.

**Methodology/Principal Findings:**

An open label controlled trial was conducted in 426 healthy Indian participants previously enrolled in a large efficacy trial. To assess whether an OCV regimen given after five years can elicit an antibody response equal to that of a primary series, we compared vibriocidal antibody titers in previously immunized participants receiving a two dose booster regimen to participants receiving a primary two dose immunization series. Among participants receiving a two dose primary series of OCV (n = 186), 69% (95% CI 62%-76%) seroconverted. In the intervention arm (n = 184), 66% (95% CI 59%-73%) seroconverted following a two dose boosting schedule given five years following the initial series. Following a single boosting dose, 71% (95% CI 64%-77%) seroconverted. Children demonstrated 79% (95% CI 69%-86%) and 82% (95% CI 73%-88%) seroconversion after primary and boosting regimens, respectively.

**Conclusions/Significance:**

Administration of an OCV boosting regimen elicits an immune response similar to those receiving a primary series in endemic areas. Though a single boosting dose induces a strong immune response, further investigations are needed to measure if these findings translate to clinical protection.

## Author Summary

The five year efficacy results of the bivalent, killed whole cell oral cholera vaccine (WC OCV) was shown to offer 65% protection in cholera endemic Kolkata. Further search strategies focused on natural boosting of immunity, since this trial assessed protection in a population that has endemic cholera at high rates every year. The efficacy demonstrated in this project reflected both vaccine and naturally induced immunity. Though efficacy is maintained for five years, no formal recommendations on a boosting regimen exist. This study suggests that a boosting regimen of killed OCV can elicit vibriocidal titers similar to those levels produced by a primary series in adults and children residing in endemic areas. A boosting recommendation could help to ease logistical challenges faced in maintaining protection in cholera endemic areas. These immunogenicity findings provide initial evidence to support the use of an OCV boosting regimen five years following the primary series, with consideration of a shorter interval for children under the age of 5 years due to a lower observed efficacy in field trials.

## Introduction

Recent outbreaks in Haiti, Pakistan, and throughout the African continent, along with increased antimicrobial resistance and the heightening awareness of climate’s role upon the global burden have contributed to renewed interest in global cholera control. Though improved water and sanitation should continue to be the mainstays of cholera-prevention efforts, major improvements are a far off goal for much of the cholera-affected developing world. The notion that cholera epidemics are short lived are refuted by the fact that outbreaks have become more frequent, larger, and longer lasting, with case fatality rates higher than four percent [1]. Many countries with endemic disease either neglect or are unable to report cases greatly due to fears of the potential impact on their economy. With about 1.4 billion people at risk for cholera, an estimated 2.8 million cases, and 91,000 deaths occurring annually, common annual incidence estimates by the World Health Organization are likely conservative [2]. The disease has become more complicated in this pandemic since the emergence of the current O1 variant El Tor biotype due to concerns of heightened virulence [3]. These new organisms are better at surviving and more likely to result in asymptomatic carriage, meaning that infection may be introduced easier into a new area unknowingly, and once present, that area may well become a new cholera endemic zone [4]. Interest in oral cholera vaccine (OCV) has increased following demonstration of protective immunity via local, mucosally secreted intestinal antibodies [5].

A large cluster randomized, double blind, placebo controlled trial was conducted in the cholera endemic urban slums of Kolkata, India in late 2006 to evaluate the protection offered by the killed bivalent OCV. Though vibriocidal titers wane by one year after dosing, a cumulative vaccine protective efficacy of 65% has been measured over five years [6,7]. Vaccine efficacy was much lower in children one to five years of age (42%). Though not completely understood, reasons for this finding may include interference with pre-existing maternal antibodies, underlying co-existing enteric infections, mucosal damage following enteropathy, and malnutrition [8]. However, it bears special mention that more cases were prevented by vaccination (10.5/1000) in the younger age group (1–5 years), compared with older age groups (5.5/1000 in 5–15 years and 3.1/1000 in ≥15 years). Significant protection of unvaccinated individuals has been demonstrated in areas of modest vaccine coverage [9]. Mathematical models based on these data suggest when vaccinating over half of the population in cholera endemic areas, incidence can be reduced by 93% due to the vaccine’s ability to induce herd protection [10]. The WHO recommends that immunization with the safe, efficacious and affordable oral cholera vaccines should be used in conjunction with other prevention and control strategies in endemic areas with a potential role in outbreak situations [11]. As final analysis of the five year efficacy results were underway, questions on how best to deploy this vaccine were being raised by national and global policy makers.

Based on the currently available efficacy data, Shanchol provides five years of clinical protection to adults in an endemic region [7]. Though no official recommendations on booster regimens are in place, redosing of the related Dukoral (killed whole cell OCV with recombinant B subunit cholera toxin) is recommended every 2 years in adults and every 6 months in young children. The aim of this trial was to measure the immunogenicity and safety of a boosting regimen five years after initial dosing in adults and children.

## Methods

### Study Design and Procedure

This is a nested, open-label controlled trial of Shanchol, an oral cholera vaccine, conducted among healthy non-pregnant subjects aged 6–14 years and ≥15 years who were initially immunized with two doses of vaccine or placebo five years before as part of a large randomized controlled trial (RCT) of an oral cholera vaccine. Because participants of the original RCT were aged ≥1 year of age, the minimal age of participants in this trial five years later was 6 years of age. All participants who were in the placebo arm of the phase III efficacy trial were scheduled to receive two doses of vaccine at the end of the trial period. Since unblinding was performed to conduct analysis and identify all individuals who needed to be given the vaccine, an open label trial design was employed in this study. The modified killed bivalent whole cell vaccine contained 1.5 x 10^11^ inactivated *V*. *cholerae* O1 and 5 x 10^10^ *V*. *cholerae* O139 bacteria consisting of: 600 ELISA units of of *V*. *cholerae* O1 El Tor Inaba; 300 ELISA units of multiple strains of *V*. *cholerae* O1 classical Ogawa, and 600 ELISA units of *V*. *cholerae* O139. Two doses of vaccine were given 2 weeks apart from May 9 to June 11, 2012. Additional details on the study site, study agents, and trial conduct for the randomized controlled trial (RCT) in Kolkata have been reported previously [12,13].

**Fig 1:**
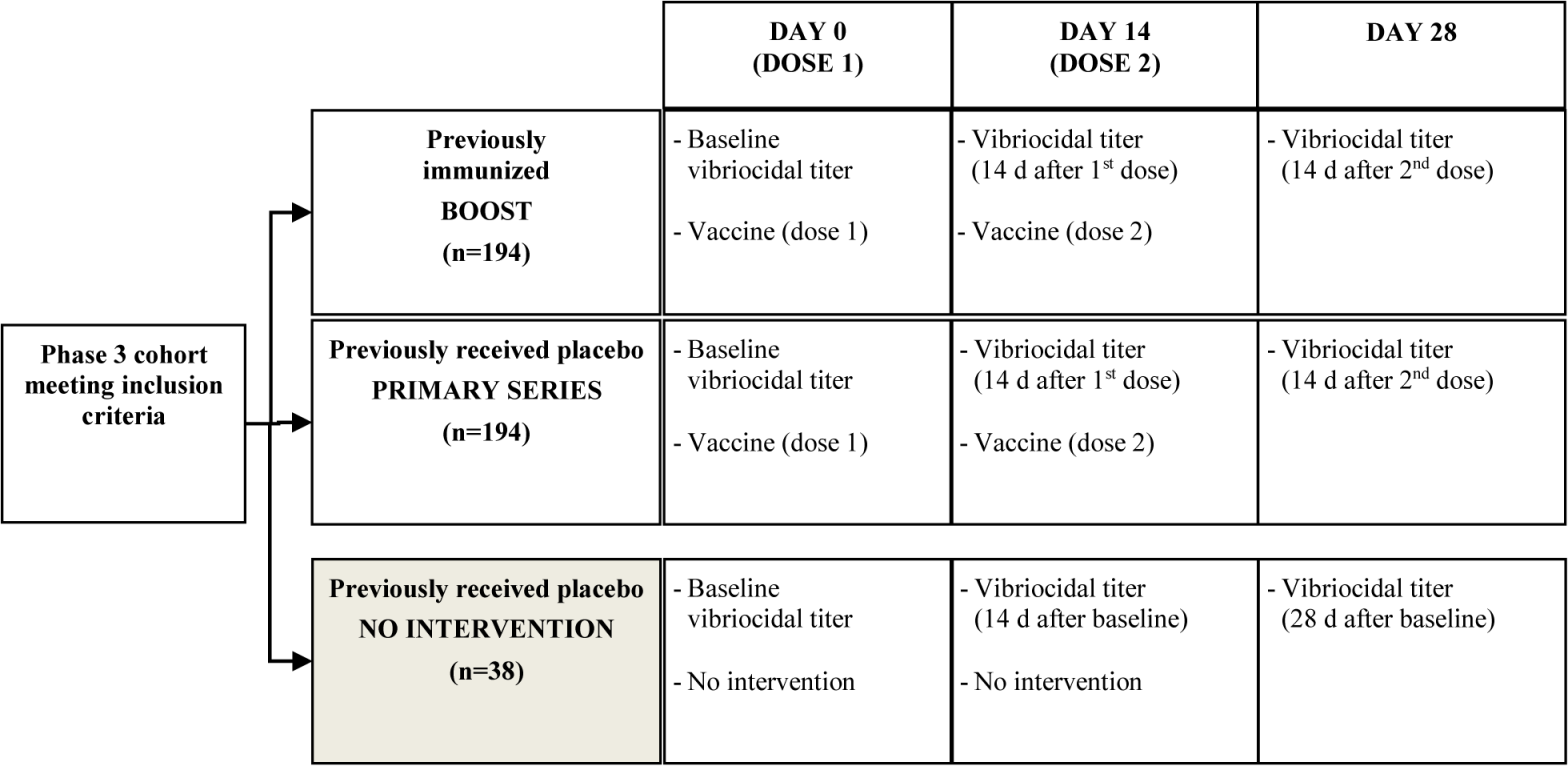
Study schedule for participants. *Follow up by healthcare provider was conducted on day 42 (28 days after the second dose) for those in the intervention groups to monitor and document any adverse or severe adverse events.

Participants were enrolled from this cohort and all follow up study activities took place at one of nine area health centers within the census area. Of the preselected trial cohort, exclusion criteria included those < six years of age, pregnant women (identified by verbal screening of married women), and individuals too weak to get out of bed, and anyone who had received vaccine following its licensure in 2009. Endpoints were compared between two intervention groups: a boosted population (individuals who received vaccine five years prior and were redosed) and a primary series population (participants who were placebo recipients in the original RCT and were receiving vaccine for the first time. Both of these groups received vaccine at days 0 and 14 and blood were drawn for measurement of vibriocidal titers. A third blood sample was also drawn on day 28 to compare baseline with titers 14 days following doses one and two (Fig 1). A documented follow up with a health care provider was performed on day 42 (28 days after the second dose in both intervention groups to monitor and document any adverse or serious adverse events. A small non-intervention arm, who did not receive vaccine, was added to ensure that boosting was not due to natural exposure. All of these individuals were given two doses of the vaccine following the final bleed of this study. Though the primary objective was to determine if a two dose OCV booster dose regimen administered to a previously immunized cohort elicits a similar immune response to those achieved by a primary immunization series, we also measured responses following a one dose booster. Seroconversion was defined as ≥ four-fold rise in serum vibriocidal titers measured at baseline (day 0) and 14 days following each dosing schedule. The previously described microtiter technique was used to detect serum vibriocidal antibodies to *V*. *cholerae* O1 El Tor Inaba, O1 Ogawa, and O139 [14].

The trial protocol was approved by the Scientific Advisory Committee and Institutional Ethics committee of the National Institute of Cholera and Enteric Diseases (NICED) and the International Vaccine Institute (IVI). Written informed consent was obtained from residents aged 18 years and older and from the parents/guardians of residents aged 1–17 years. Written assent was also obtained from residents aged 12–17 years. The trial was registered with Clinical Trial Registry-India (CTRI/2012/04/002574) and ClinicalTrials.gov (NCT01579448). The funding agencies of the study had no role in study design, data collection, data analysis, data interpretation, or writing of the report

### Subject Allocation

Potential enrollees in each arm were identified before-hand by the IVI biostatistics team. All eligible individuals consist of those who received two doses of study agent during the RCT in September 2006. Furthermore, eligible subjects were verified to be present in the census conducted in the fifth year of the efficacy trial. Any participant diagnosed with cholera during the five year surveillance period was not included. Participants were stratified according to the size of each of the nine health center catchment areas. Randomly generated lists for each stratum were made, from which community health workers contacted and approached potential participants during times of community sensitization of this new project.

### Statistical Methods

Sample size calculations were based on the non-inferiority of the primary intervention (re-immunization with 2 booster doses in previously immunized) compared to two doses of vaccine in an unimmunized population. Using 80% power, 65% immune response, a non-inferiority limit of 20%, and 10% dropout rate, we would require 194 participants (97 children ages 6–14 and 97 adults ages ≥15) in each of the two arms. To ensure that natural infection with *V*. *cholerae* did not affect serum antibody responses, a smaller non-intervention arm was recruited. Assuming an 80% power, a 5% immune response, and 10% drop out, to accept >20% immune response in intervention arm, we would require 38 participants (18 children ages 6–14 and 17 adults ages ≥15) in the non-intervention arm. Demonstration of a fourfold or greater rise in serum anti-O1 vibriocidal antibody titer following the second dose was the primary endpoint of immunogenicity. Comparison of the primary endpoint, vibriocidal seroconversion in boosting and primary series arms were evaluated with one-tailed 97.5% confidence interval using the Wilson Score method [15]. The dichotomous variables were compared using the chi-square test or by the Fisher’s exact test if a predicted cell count is less than five. For dimensional variables such as vibriocidal titers, Student’s t-test or Satterthwaite method depending on the heterogeneity of variance were used. Vibriocidal titers and fold rises were logarithmically transformed prior to statistical analyses. Statistical significance threshold of all comparisons was set at p<0.05 and two-tailed. All statistical analyses were done with SAS version 9.3.

## Results

### Participant Recruitment and Baseline Data

**Fig 2:**
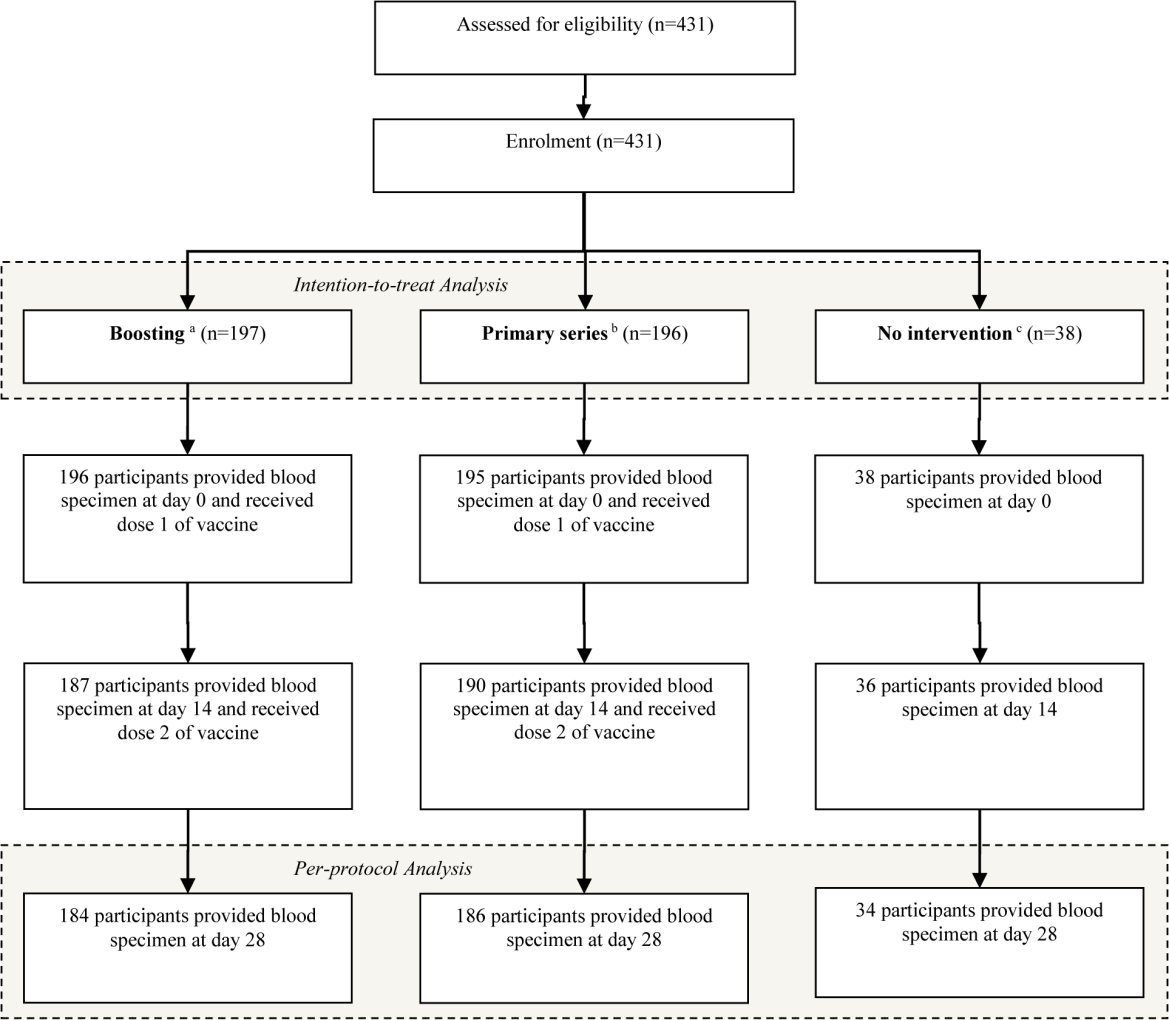
Flowchart of adult and children participants in the study. ^a^ One subject didn’t provided blood specimen at day 0 and 12 subjects temporally migrated out (9 subjects at day 14 and 3 subjects at day 28) during the period in Boosting group. ^b^ One subject didn’t provided blood specimen at day 0 and 9 subjects temporally migrated out (5 subjects at day 14 and 4 subjects at day 28) during the period in Primary Series group. ^c^ Four subjects temporally migrated out (2 subjects at day 14 and 2 subjects at day 28) during the period in the No intervention group.

Recruitment and follow up was conducted in May-June 2012 and participant flow is illustrated in Fig 2. Among eligible participants in the intervention groups, 184/197 (97%) enrollees of the booster arm and 186/196 (95%) of the primary immunization arm took both doses and provided all three blood samples. A total of 27 participants (6%) were lost to follow up or considered ineligible following screening. There were no major differences between groups with regards to key demographic indicators (Table 1).

**Table 1:** Demographic characteristics of study participants.

Characteristics	Boosting n = 197 (%)	Primary series n = 196 (%)	No intervention n = 38 (%)	*p value (Boost vs Primary)*
Sex: Male	95 (48.0)	94 (48.0)	16 (42.0)	0.96
Mean age in years	24.4	25.6	26.5	0.50
Literate household head	130 (66.0)	130 (66.0)	29 (76.0)	0.94
Flush toilet used alone	8 (4.1)	16 (8.2)	1 (2.6)	0.09
Own tap water for drinking	21 (10.7)	30 (15.3)	5 (13.2)	0.17
Boiled/Filtered water generally	15 (7.6)	19 (9.7)	2 (5.3)	0.46
Always wash hands with water and soap after defecation	142 (72.1)	133 (67.9)	22 (57.9)	0.36
Owned house	63 (32.0)	64 (32.7)	6 (15.8)	0.89

### Outcomes

A per protocol analysis was conducted for all immunogenicity data. Baseline geometric mean titers against *V*. *cholerae* O1 Inaba (GMT, 95% CI) ranged from 103 to 183 in the previously immunized group compared to a range 70 to 125 in those receiving OCV as a primary series. Baseline titers to O1 Inaba were higher in the previously immunized groups but the difference was not significant (Table 2). Geometric fold rise (GFR) following two doses ranged from 6 to 10 in both intervention groups. Seroconversion rates to O1 Inaba were 66% [95% CI 59%-73%] and 69% [95% CI 62%-76%] following two dose regimens in the boosting and primary immunization arms (p = 0.53), indicating that the boosting arm was non-inferior to the primary immunization arm. Seroconversion of 71% following a one dose regimen in the boosting arm was non-inferior to 69% after a two dose regimen in the primary immunization arm. When comparing the immune responses to O1 Inaba, there was no significant difference in the geometric mean fold rise or the percentage who seroconverted in the boosting and primary arms following two doses of OCV (82% [95% CI 73%-88%] vs 79% [95% CI 69%-86%] in the 6–14 year age group and 51% [95% CI 40%-61%] vs 60% [95% CI 50%-70%] in ≥15 years age group). While the GMT of the boosting arm in children was double that of the primary series after the first dose, the GMFr was also found to be high in both arms. In contrast, adults demonstrated similar GMT in both arms. Because of the vaccine’s lower efficacy in the youngest group, analysis was further subdivided into a 6–10 year group (1–5 year age cohort in the previous efficacy trial) [S1 Table]. Similar to the 6–14 year age group, the 6–10 year group demonstrated similar baseline GMT in boosting and primary groups, with significantly higher GMTs following the first (2642 vs 931, p = 0.01) and second dose (1201 vs 534, p = 0.02) against *V*. *cholerae* O1 Inaba. Likewise, both groups also showed no significant differences in geometric fold rises or percent serconversion following the first or second dose.

**Table 2:** Serum vibriocidal antibody titers and proportion of ≥4 fold rise from baseline GMT to *V. cholerae* O1 Inaba in all ages (≥ 6 years).

***V. cholerae* O1 Inaba ≥ 6 years old**	**Day 0 (Baseline)**	**Day 14 (Dose 1)**			**Day 28 (Dose 2)**		
	GMT^a^ (95% CI)	GMT^a^ (95% CI)	GMF rise^b^ (95% CI)	Serocon-version^c^ (95% CI)	GMT^a^ (95% CI)	GMF rise^b^ (95% CI)	Serocon-version^c^ (95% CI)
Boosting (n = 184)	137 (103,183)	1859 (1532, 2255)	13.5 (10,18)	71% (64%, 77%)	1072 (900, 1276)	7.8 (6,10)	66% (59%, 73%)
Primary series (n = 186)	94 (70,125)	1233 (1000, 1521)	13 (10,17)	78% (72%, 84%)	744 (617, 896)	7.9 (6,10)	69% (62%, 76%)
No Intervention (n = 34)	56 (27,120)	55 (28,110)	1 (0.8, 1.1)	3% (1%, 15%)	54 (27,108)	0.9 (0.8, 1.1)	6% (2%, 19%)
*p* value (Boost *v* Primary)	0.06	<0.01	0.89	0.11	<0.01	0.93	0.53
Proportion difference (95% CI)				-7% (-16%, 1%)^d^			-3% (-12%, 6%)^e^
***V*. *cholerae O1 Inaba 6–14 years old***	**Day 0 (Baseline)**	**Day 14 (Dose 1)**			**Day 28 (Dose 2)**		
	GMT^a^ (95% CI)	GMT^a^ (95% CI)	GMF rise^b^ (95% CI)	Serocon-version^c^ (95% CI)	GMT^a^ (95% CI)	GMF rise^b^ (95% CI)	Serocon-version^c^ (95% CI)
Boosting (n = 93)	80.6 (52,124)	2657 (2071, 3410)	33 (21,51)	85% (76%, 91%)	1319 (1048, 1659)	16.4 (11, 24)	82% (73%, 88%)
Primary (n = 90)	49.6 (32,75)	1270 (882, 1829)	25.6 (17,38)	88% (79%, 93%)	693 (504, 952)	13.9 (10,20)	79% (69%, 86%)
No Intervention (n = 18)	26.2 (10, 65)	28.3 (13, 63)	1.1 (0, 1.4)	6% (1%, 26%)	28.3 (12, 65)	1.1 (0.8, 1.4)	11% (3%, 33%)
*p* value (Boost *vs* Primary)	0.11	<0.01	0.4	0.58	<0.01	0.56	0.63
Proportion difference (95% CI)				-3% (-13%, 8%)^d^			3% (-9%, 15%)^e^
***V*. *cholerae O1 Inaba ≥ 15 years old***	**Day 0 (Baseline)**	**Day 14 (Dose 1)**			**Day 28 (Dose 2)**		
	GMT^a^ (95% CI)	GMT^a^ (95% CI)	GMF rise^b^ (95% CI)	Serocon-version^c^ (95% CI)	GMT^a^ (95% CI)	GMF rise^b^ (95% CI)	Serocon-version^c^ (95% CI)
Boosting (n = 91)	237.8 (168, 337)	1290 (973, 1710)	5.4 (4, 7)	57% (47%, 67%)	868 (669, 1126)	3.6 (3, 5)	51% (40%, 61%)
Primary (n = 96)	170.7 (119, 245)	1199 (957, 1504)	7 (5, 9)	70% (60%, 78%)	795 (645,980)	4.6 (3, 6)	60% (50%, 70%)
No Intervention (n = 16)	134.5 (41, 438)	118 (38,362)	0.9 (0.7, 1.1)	0%	113 (37,347)	0.8 (0.7, 0.9)	0%
*p* value (Boost *vs* Primary)	0.19	0.69	0.28	0.07	0.6	0.25	0.17
Proportion difference (95% CI)				-13% (-26%, 1%)^d^			-10% (-24%, 4%)^e^

^a^Geometric mean reciprocal titers.

^b^Geometric mean-fold rise from baseline to 14 days post first vaccine dose or from baseline to 14 days post second vaccine dose.

^c^Percent of subjects with ≥ 4 fold rise in titers from baseline to 14 days post first vaccine dose or from baseline to 14 days post second vaccine dose. 95% confidence intervals derived using Wilson Score method.

^d^Difference seroconversion rates (95% CI) after single dose are calculated by subtracting those following primary series from those following booster dose. In the aged ≥ 6 years old group, seroconversion rates following single booster dose (71%) is noninferior than those following one dose of a primary series (78%) as the lower limit of the proportion difference is greater than clinical margin (-20%)

^e^Difference seroconversion rates (95% CI) after two doses are calculated by subtracting those following primary series from those following booster doses. In the aged ≥ 6 years old group, seroconversion rates following two booster doses (66%) is noninferior than those following two doses primary series (69%) as the lower limit of the proportion difference is greater than clinical margin (-20%)

Furthermore, seroconversion rates to *V*. *cholerae* O1 Inaba following a single booster dose (85% in 6–14 years, 57% in ≥15 years) were comparable to those following a two dose primary series (79% in 6–14 years, 60% in ≥15 years). While seroconversion rates following one and two booster doses (57% and 51%) are not significantly different from one and two doses of a primary series (70% and 60%) in volunteers >15 years of age, the results regarding non-inferiority are inconclusive since the 95% CI of the proportion difference includes the clinical margin (-20%) and zero. There was no significant rise in seroconversion rates in the non-intervention arm (11% in 6–14 years, 0% in ≥15 years), supporting the observation that environmental exposure was not a major determinant in seroconversion, as compared to vaccine in accounting for the rises in seroconversion rates in the intervention arms.

Vibriocidal responses to O1 Ogawa demonstrated no significant difference in the percentage seroconversion in the boosting or primary arms following two doses of OCV (66% [95% CI 55%-74%] vs 72% [95% CI 62%-80%] in the 6–14 year age group and 41% [95% CI 31%-51%] vs 53% [95% CI 43%-63%] in ≥15 years age group). Though seroconversion rates for *V*. *cholerae* O1 Ogawa were significantly higher in the primary series after one dose (73% v 63% *p 0*.*03)*, this could be explained by the significantly higher baseline GMTs observed between the two intervention groups. No significant difference was noted when comparing the boosting regimen to the two dose primary (53% vs 62% *p = 0*.08) (Table 3). Because each study population arm differed in their previous antigenic exposure, we also analyzed vibriocidal response based upon median baseline geometric mean titer for each age group. Geometric mean fold rise and seroconversion rates following two doses did not significantly vary between boosting and primary arms measuring response against *V*.*cholerae* O1 Inaba and O1 Ogawa (Table 4). Our analysis notes the recurrent observation of lower immune responses to O139 (S2 Table), likely representing that Shanchol does not provide meaningful sero-responses against O139, at least as measured by vibriocidal responses.

**Table 3:** Serum vibriocidal antibody titers and proportion of ≥4 fold rise from baseline GMT to *V. cholerae* O1 Ogawa in all ages (≥ 6 years).

***V*. *cholerae O1 Ogawa* ≥ 6 years old**	**Day 0 (Baseline)**	**Day 14 (Dose 1)**			**Day 28 (Dose 2)**		
	GMT^a^ (95% CI)	GMT^a^ (95% CI)	GMF rise^b^ (95% CI)	Serocon-version^c^ (95% CI)	GMT^a^ (95% CI)	GMF rise^b^ (95% CI)	Serocon-version^c^ (95% CI)
Boosting (n = 184)	333.5 (255, 436)	2771 (2319, 3311)	8.3 (6.3, 11)	63% (55%, 69%)	1730 (1474, 2031)	5.2 (4.1, 6.6)	53% (46%, 60%)
Primary series (n = 186)	186 (137, 253)	2093 (1772, 2473)	11.2 (8.5, 14.8)	73% (66%, 79%)	1437 (1240, 1664)	7.7 (5.9, 10)	62% (55%, 69%)
No Intervention (n = 34)	142.6 (62.5, 321)	138.7 (64.2, 300)	1 (0.8, 1.1)	0%	138.7 (64, 301)	1 (0.8, 1.1)	0%
p value (Boost vs Primary)	0.01	0.02	0.13	0.03	0.09	0.03	0.08
Proportion difference (95% CI)				-11% (-20%, -1%)^d^			-9% (-19%, 1%)^e^
***V*. *cholerae O1 Ogawa 6–14 years old***	**Day 0 (Baseline)**	**Day 14 (Dose 1)**			**Day 28 (Dose 2)**		
	GMT^a^ (95% CI)	GMT^a^ (95% CI)	GMF rise^b^ (95% CI)	Serocon-version^c^ (95% CI)	GMT^a^ (95% CI)	GMF rise^b^ (95% CI)	Serocon-version^c^ (95% CI)
Boosting (n = 93)	235.7 (152, 365)	3688 (2900, 4691)	15.6 (10, 24.4)	70% (60%, 78%)	2125 (1712, 2637)	9 (6, 13.4)	66% (55%, 74%)
Primary (n = 90)	101.6 (63, 163.8)	2263 (1755, 2919)	22.3 (14.9, 33.4)	88% (79%, 93%)	1361 (1098, 1688)	13.4 (8.8, 202)	72% (62%, 80%)
No Intervention (n = 18)	56.6 (19.7, 162)	58.8 (20.9, 165)	1 (0.9, 1.2)	0%	58.8 (21.7, 159.5)	1 (0.8, 1.2)	0%
p value (Boost vs Primary)	0.01	0.01	0.25	0.003	<0.001	0.17	0.333
Proportion difference (95% CI)				-18% (-29%, -6%)^d^			-7% (-20%, 7%)^e^
***V*. *cholerae O1 Ogawa ≥ 15 years old***	**Day 0 (Baseline)**	**Day 14 (Dose 1)**			**Day 28 (Dose 2)**		
	GMT^a^ (95% CI)	GMT^a^ (95% CI)	GMF rise^b^ (95% CI)	Serocon-version^c^ (95% CI)	GMT^a^ (95% CI)	GMF rise^b^ (95% CI)	Serocon- version^c^ (95% CI)
Boosting (n = 91)	475 (353, 640)	2068 (1605, 2665)	4.3 (3.3, 5.8)	55% (45%, 65%)	1403 (1110, 1772)	2.9 (2.3, 3.7)	41% (31%, 51%)
Primary (n = 96)	329.4 (229.7, 472.3)	1946 (1560, 2427)	5.9 (4.2, 8.3)	59% (49%, 69%)	1511 (1232, 1854)	4.6 (3.4, 6.2)	53% (43%, 63%)
No Intervention (n = 16)	397.4 (122.9, 1285)	364.4 (125.8, 1056)	0.9 (0.7, 1.2)	0%	364.4 (119.6, 1110)	0.9 (0.7, 1.2)	0%
p value (Boost vs Primary)	0.12	0.72	0.17	0.54	0.63	0.03	0.09
Proportion difference (95% CI)				-4% (-18%, 10%)^d^			-12% (-26%, 2%)^e^

^a^Geometric mean reciprocal titers.

^b^Geometric mean-fold rise from baseline to 14 days post first vaccine dose or from baseline to 14 days post second vaccine dose.

^c^Percent of subjects with ≥ 4 fold rise in titers from baseline to 14 days post first vaccine dose or from baseline to 14 days post second vaccine dose. 95% confidence intervals derived using Wilson Score method.

^d^Difference seroconversion rates (95% CI) after single dose are calculated by subtracting those following primary series from those following booster dose

^e^Difference seroconversion rates (95% CI) after two doses are calculated by subtracting those following primary series from those following booster doses

**Table 4:** Geometric mean fold rises to *V. cholerae* O1 Inaba and Ogawa, and number who develop ≥ 4 fold rises from baseline after two doses.

***V*. *cholerae* O1 Inaba**	**GMF—rise from baseline** ^**a**^			**No. with ≥ 4 fold rise from baseline** ^**b**^		
	**Boosting**	**Primary**	**p value** ^**c**^	**Boosting**	**Primary**	**p value** ^**d**^
**All ages (≥ 6 years)**						
All (n = 370)	7.8 (n = 184)	7.9 (n = 186)	0.77	122/184 (66.3)	129/186 (69.4)	0.61
Baseline vibriocidal ≤ 160 (n = 214)	20.7 (n = 100)	17.8 (n = 114)	0.56	87 /100 (87.0)	101/114 (88.6)	0.88
Baseline vibriocidal > 160 (n = 156)	2.4 (n = 84)	2.2 (n = 72)	0.76	35 /84 (41.7)	28 /72 (38.9)	0.85
**6–14 years**						
All (n = 183)	16.4 (n = 93)	14.0 (n = 90)	0.71	76 /93 (81.7)	71 /90 (78.9)	0.77
Baseline vibriocidal ≤ 80 (n = 106)	52.5 (n = 49)	32.1 (n = 57)	0.13	48 /49 (98.0)	53 /57 (93.0)	0.37
Baseline vibriocidal > 80 (n = 77)	4.5 (n = 44)	3.3 (n = 33)	0.27	28 /44 (63.6)	18 /33 (54.5)	0.57
**≥ 15 years**						
All (n = 187)	3.7 (n = 91)	4.7 (n = 96)	0.26	46 /91 (50.5)	58 /96 (60.4)	0.23
Baseline vibriocidal ≤ 320 (n = 126)	6.2 (n = 58)	7.4 (n = 68)	0.68	40 /58 (69.0)	52 /68 (76.5)	0.46
Baseline vibriocidal > 320 (n = 61)	1.5 (n = 33)	1.5 (n = 28)	0.52	6 /33 (18.2)	6 /28 (21.4)	1.00
***V*. *cholerae* O1 Ogawa**	**GMF—rise from baseline** ^**a**^			**No. with ≥ 4 fold rise from baseline** ^**b**^		
	**Boosting**	**Primary**	**p value** ^**c**^	**Boosting**	**Primary**	**p value** ^**d**^
**All ages (≥ 6 years)**						
All (n = 370)	5.2 (n = 184)	7.7 (n = 186)	0.03	98 /184 (53.3)	116/186 (62.4)	0.10
Baseline vibriocidal ≤ 160 (n = 149)	23.5 (n = 65)	33.9 (n = 84)	0.22	57 /65 (87.7)	81 /84 (96.4)	0.06
Baseline vibriocidal > 160 (n = 221)	2.3 (n = 119)	2.3 (n = 102)	0.71	41 /119 (34.5)	35 /102 (34.3)	1.00
**6–14 years**						
All (n = 183)	9.0 (n = 93)	13.4 (n = 90)	0.15	61 /93 (65.6)	65 /90 (72.2)	0.42
Baseline vibriocidal ≤ 80 (n = 73)	61.2 (n = 31)	65.1 (n = 42)	0.86	30 /31 (96.8)	42 /42 (100.0)	0.43
Baseline vibriocidal > 80 (n = 110)	3.5 (n = 62)	3.4 (n = 48)	0.96	31 /62 (50.0)	23 /48 (47.9)	0.98
**≥ 15 years**						
All (n = 187)	2.9 (n = 91)	4.6 (n = 96)	0.05	37 /91 (40.7)	51 /96 (53.1)	0.12
Baseline vibriocidal ≤ 320 (n = 91)	5.7 (n = 38)	10.0 (n = 53)	0.11	25 /38 (65.8)	43 /53 (81.1)	0.16
Baseline vibriocidal > 320 (n = 96)	1.8 (n = 53)	1.8 (n = 43)	0.79	12 /53 (22.6)	8 /43 (18.6)	0.82

^a^ Geometric mean fold (GMF) rise from baseline to 14 days after dose 2

^b^ Number of participants with ≥ 4 fold rise in titers from baseline to 14 days after dose 2

^c^ p values comparing GMF-rise from baseline to 14 days after dose 2 between Boost and Primary groups

^d^ p values comparing ≥4-fold rise from baseline to 14 days after dose 2 between Boost and Primary groups

A total of six adverse events were recorded in the boosting arm (fever, diarrhea, abdominal pain, vertigo) and seven adverse events in the primary series arm within three days of either dose (fever, diarrhea, vertigo, body ache). All were mild and resolved with symptomatic treatment. No serious adverse events were reported within 28 days of dosing.

## Discussion

The data suggests that repeating the immunization series to an endemic population previously immunized five years prior can induce a strong vibriocidal response, meeting those of similar individuals receiving the vaccine series for the first time. Additionally, a single booster dose achieves levels as high as a two dose OCV primary series. Before summarizing the findings of this study, study limitations should be considered. We did not measure mucosal antibody responses. These are important indicators of immunity that would provide a broader understanding of vaccine response. Still, vibriocidal antibodies are thought to provide a surrogate of protection and add to our understanding of vaccine-induced immunity. Our data provides information on immune responses and adverse events following immunization. These results do not provide information on vaccine efficacy following a boost vs. primary vaccination. Observational studies evaluating one or two dose regimens at five year would, however, provide estimates of booster effectiveness. Finally, our study was conducted in an area were V. cholerae exposure is frequent. Our results may not correlate with immune responses in areas where cholera incidence is lower.

It is important to consider unique aspects of local immune responses from the gut subsequent to oral immunization. Mucosally induced antibody secreting cells (ASCs) have been shown to migrate into the peripheral circulation, where responses in naturally primed individuals are appreciably quicker than non-primed subjects [16]. Investigators in Sweden have reported substantial ASC responses after a single booster dose over 10 years later, which were highest at 4–5 days and followed by a rapid decline [17]. Much like vibriocidal antibody responses, a second dose of OCV given on day 14 did not boost ASC in Bangladesh [18], whereas OCV given at the same schedule in a non-endemic areas [19]. These findings support the presence of a long term memory response that may support an extended gap when considering recommended intervals for a boosting regimen. Furthermore, boosting strategies likely differ between areas where *V*. *cholerae* infection is historically endemic and those where it is not, suggesting that one dose of OCV may offer some degree of immediate protection in primed populations living in cholera endemic areas.

Though vibriocidal antibody response reflects an indirect correlate of protection, the public health implications of these findings could provide a basis to improve implementation of delivering OCV in resource constrained settings. When factoring these results in combination with recent studies expressing longer duration of efficacy [7] and the possibility of a flexible dosing regimen[20], this boosting data may extend the true ‘benefit horizon’ of this affordable and feasible vaccine. These findings have particular relevance for endemic countries, for which longstanding protection is aided by natural boosting via regular environmental exposure. In light of limited oral cholera vaccine supply, the possibility of a one dose booster regimen would increase the number of individuals for which vaccine is available in endemic areas. If an OCV can provide long lasting clinical protection, capitalizing on the ease of delivery and immunological advantages of using the mucosal port of entry, a boosting regimen at five years in endemic populations could serve to trigger an immune response. Because baseline vibriocidal antibodies and memory to the cholera pathogen already exist in endemic populations, a boosting regimen could raise antibody production, potentially offering protection to a naturally primed population. Pre-existing immunity plays a critical role in understanding the host defense of each unique host population. Investigations on memory B cell and cell-mediated immune responses are lacking in children, and such studies would be interesting to offer important insights into differences in protection offered by natural infection versus current vaccine options [21]. However, our understanding of this phenomenon is limited in less endemic populations, and should be one of the priorities for future OCV field evaluations.

As demonstrated in this study and previous trials, the antibody response after the first of two doses are higher than after the second dose [22], implying that an immune response may begin even before the second dose is administered. Though vibriocidal antibodies have been shown to wane one year following dosing, clinical protection has been maintained for five years in an endemic setting [6,7]. Part of this vaccine’s success may have been attributable to natural boosting in a highly endemic context, as in the urban slum populations of Kolkata. Recurring cholera exposures can lead to a progressive age related acquisition of natural immunity due to environmental boosting and memory B cell mediated anamnestic responses [21]. This trial demonstrates that a two dose boosting OCV boosting regimen results in a robust immune response. Because it stimulates vibriocidal titers comparable to those achieved in residents receiving a full primary series of OCV, this data could serve as the base for future investigations examining clinical protection offered by an OCV boosting regimen. A shorter interval may likely be considered in children under five years of age due the lower cumulative protective efficacy (42%) in this age group, Complementary efforts to strengthen effective surveillance are vital in order to accurately assess the impact of any new dosing strategy. With proper disease detection programs in place and supporting epidemiologic data, this evidence could support the initiation of a boosting regimen policy recommendation.

Large cholera outbreaks continue to threaten marginalized populations affected by natural disasters or those displaced by war, where there is inadequate sewage disposal and contaminated water. It remains a major international public health priority and is a risk to most developing countries. Following introduction of *V*. *cholerae* to previously non-endemic low income countries with weak water and sanitation infrastructure, the delineation of endemic and epidemic is becoming less defined. With the recent support to build a 20 million dose OCV stockpile by 2018 [23], appropriate boosting strategies need to be considered by policy makers now to successfully prevent future recurrences.

## Supporting Information

S1 Table Serum vibriocidal antibody titers and proportion of ≥4 fold rise from baseline GMT to *V*. *cholerae* O1 Inaba, Ogawa, and O139 in aged 6–10 years old. (DOCX)

S2 Table Serum vibriocidal antibody titers and proportion of ≥4 fold rise from baseline GMT to *V*. *cholerae* O139 in all ages (≥ 6 years). (DOCX)

S1 Checklist Consort checklist page 1. (PDF)

S2 Checklist Consort checklist page 2. (PDF)
