# Supplementary material for: An Open Label Non-inferiority Trial Assessing Vibriocidal Response of a Killed Bivalent Oral Cholera Vaccine Regimen following a Five Year Interval in Kolkata, India
Source: PLoS Negl Trop Dis. 2015 May 29;9(5):e0003809. doi: 10.1371/journal.pntd.0003809 (PMC4449043; doi:10.1371/journal.pntd.0003809)
Supplement: S1 Table — (DOCX) [file pntd.0003809.s001.docx]

**Table S1: Serum vibriocidal antibody titers and proportion of ≥4 fold rise from baseline GMT to V. cholerae O1 Inaba, Ogawa, and O139 in aged 6-10 years old**

| ***V. cholerae O1 Inaba***  ***6-10 years old*** | **Day 0 (Baseline)** | **Day 14**  **(Dose 1)** | | | | **Day 28**  **(Dose 2)** | | |
| --- | --- | --- | --- | --- | --- | --- | --- | --- |
|  | GMT^a^  (95% CI) | GMT^a^  (95% CI) | GMF rise^b^  (95% CI) | Serocon-version^c^  (95% CI) | | GMT^a^  (95% CI) | GMF rise^b^  (95% CI) | Serocon-version^c^  (95% CI) |
| Boosting (n=44) | 49.9  (25.8, 96.3) | 2642  (1832, 3811) | 53.0  (26.9,104.3) | 89%  (76%, 95%) | | 1221  (860.1, 1733) | 24.5  (13.9, 43.1) | 89%  (76%, 95%) |
| Primary series (n=37) | 35.1  (17.9, 68.6) | 930.9  (514.4, 1685) | 26.5  (14.2, 49.6) | 89%  (75%, 96%) | | 533.9  (315.3,903.9) | 15.2  (8.1, 28.8) | 78%  (63%, 89%) |
| No Intervention (n=6) | 25.2  (4.9,129.6) | 25.2 (6.0,105.3) | 1.0  (0.6, 1.6) | 0% | | 28.3  (4.8,165.5) | 1.1  (0.5, 2.3) | 17%  (3%, 56%) |
| *p* value (Boost *v* Primary) | 0.48 | 0.01 | 0.14 | 1 | | 0.02 | 0.29 | 0.34 |
| Proportion difference (95% CI) |  |  |  | -1%  (-16%, 14%)^d^ | |  |  | 10%  (-7%, 27%)^e^ |
| ***V. cholerae O1 Ogawa***  ***6-10 years old*** | **Day 0 (Baseline)** | **Day 14**  **(Dose 1)** | | | | **Day 28**  **(Dose 2)** | | |
|  | GMT^a^  (95% CI) | GMT^a^  (95% CI) | GMF rise^b^  (95% CI) | Serocon-version^c^  (95% CI) | | GMT^a^  (95% CI) | GMF rise^b^  (95% CI) | Serocon-version^c^  (95% CI) |
| Boosting (n=44) | 157.5  (78.9,314.5) | 4042  (2932, 5574) | 25.7  (12.8, 51.7) | 82%  (68%, 90%) | | 1959  (1433, 2676) | 12.4  (6.5, 23.7) | 73%  (58%, 84%) |
| Primary (n=37) | 83.1  (40.2,171.8) | 2083  (1343, 3231) | 25.1  (13.9, 45.1) | 92%  (79%, 97%) | | 1233  (877.6, 1732) | 14.8  (7.8, 28.2) | 76%  (60%, 87%) |
| No Intervention (n=6) | 100.8  (9.0, 1133) | 113.1  (9.6, 1333) | 1.1  (0.8, 1.5) | 0% | | 100.8  (9.8, 1037) | 1.0  (0.6, 1.6) | 0% |
| *p* value (Boost *vs* Primary) | 0.18 | 0.02 | 0.77 | 0.32 | | 0.05 | 0.6 | 0.96 |
| Proportion difference (95% CI) |  |  |  | -10%  (-25%, 5%)^d^ | |  |  | -3%  (-22%, 16%)^e^ |
| ***V. cholerae O139***  ***6-10 years old*** | **Day 0 (Baseline)** | **Day 14**  **(Dose 1)** | | | | **Day 28**  **(Dose 2)** | | |
|  | GMT^a^  (95% CI) | GMT^a^  (95% CI) | GMF rise^b^ (95% CI) | | Serocon-version^c^  (95% CI) | GMT^a^  (95% CI) | GMF rise^b^  (95% CI) | Serocon-version^c^  (95% CI) |
| Boosting (n=43) | 4.3  (3.2, 5.8) | 16.4  (10.7, 25.1) | 3.8  (2.6, 5.7) | | 48%  (34%, 62%) | 11.8  (8.0, 17.4) | 2.8  (1.9, 3.9) | 43%  (30%, 58%) |
| Primary (n=37) | 4.3  (3.1, 6.1) | 15.0  (8.8, 25.6) | 3.5  (2.1, 5.7) | | 46%  (31%, 62%) | 10.8  (7.3, 16.2) | 2.5  (1.8, 3.6) | 35%  (22%, 51%) |
| No Intervention (n=6) | 2.8  (2.1, 3.8) | 3.4  (1.6, 7.3) | 1.2  (0.8, 1.9) | | 0% | 4.2  (1.8, 10.2) | 1.5  (0.7, 3.3) | 17%  (3%, 56%) |
| *p* value (Boost *vs* Primary) | 0.96 | 0.78 | 0.65 | | 0.97 | 0.86 | 0.6 | 0.55 |
| Proportion difference (95% CI) |  |  |  | | 2%  (-20%, 23%)^d^ |  |  | 8%  (-13%, 29%)^e^ |

^a^Geometric mean reciprocal titers.

^b^Geometric mean-fold rise from baseline to 14 days post first vaccine dose or from baseline to 14 days post second vaccine dose.

^c^Percent of subjects with ≥ 4 fold rise in titers from baseline to 14 days post first vaccine dose or from baseline to 14 days post second vaccine dose. 95% confidence intervals derived using Wilson Score method.

^d^Difference seroconversion rates (95% CI) after single dose are calculated by subtracting those following primary series from those following booster dose

^e^Difference seroconversion rates (95% CI) after two doses are calculated by subtracting those following primary series from those following booster doses
