# Supplementary material for: An Open Label Non-inferiority Trial Assessing Vibriocidal Response of a Killed Bivalent Oral Cholera Vaccine Regimen following a Five Year Interval in Kolkata, India
Source: PLoS Negl Trop Dis. 2015 May 29;9(5):e0003809. doi: 10.1371/journal.pntd.0003809 (PMC4449043; doi:10.1371/journal.pntd.0003809)
Supplement: S2 Table — (DOCX) [file pntd.0003809.s002.docx]

**Table S2: Serum vibriocidal antibody titers and proportion of ≥4 fold rise from baseline GMT to V. cholerae O139 in all ages (≥ 6 years)**

| ***V. cholerae O139***  **≥ 6 years old** | **Day 0 (Baseline)** | **Day 14**  **(Dose 1)** | | | **Day 28**  **(Dose 2)** | | |
| --- | --- | --- | --- | --- | --- | --- | --- |
|  | GMT^a^  (95% CI) | GMT^a^  (95% CI) | GMF rise^b^  (95% CI) | Serocon-version^c^  (95% CI) | GMT^a^  (95% CI) | GMF rise^b^  (95% CI) | Serocon-version^c^  (95% CI) |
| Boosting (n=184) | 4.7  (4, 5.6) | 13.2  (10.7, 16.2) | 2.8  (2.3, 3.3) | 39%  (32%, 46%) | 10.7  (8.8, 13.1) | 2.3  (1.9, 2.7) | 32%  (26%, 39%) |
| Primary series (n=186) | 5.7  (4.8, 6.8) | 14.7  (11.8, 18.2) | 2.6  (2.1, 3.1) | 38%  (31%, 45%) | 13.3  (11, 16) | 2.3  (2, 2.7) | 34%  (27%, 41%) |
| No Intervention (n=34) | 5.2  (3.2, 8.3) | 5.5  (3.5, 8.6) | 1.1  (0.9, 1.3) | 3%  (1%, 15%) | 6  (3.8, 9.4) | 1.2  (0.9, 1.5) | 9%  (3%, 23%) |
| *p* value (Boost *vs* Primary) | 0.12 | 0.47 | 0.52 | 0.94 | 0.12 | 0.86 | 0.71 |
| Proportion difference (95% CI) |  |  |  | 0.4%  (-9%, 10%)^d^ |  |  | -2%  (-11%, 8%)^e^ |
|  | | | | | | | |
| ***V. cholerae O139***  ***6 – 14 years old*** | **Day 0 (Baseline)** | **Day 14**  **(Dose 1)** | | | **Day 28**  **(Dose 2)** | | |
|  | GMT^a^  (95% CI) | GMT^a^  (95% CI) | GMF rise^b^ (95% CI) | Serocon-version^c^  (95% CI) | GMT^a^  (95% CI) | GMF rise^b^  (95% CI) | Serocon-version^c^  (95% CI) |
| Boosting (n=93) | 4.2  (3.4, 5.2) | 17.5  (13.3, 23.1) | 4.2  (3.2, 5.5) | 54%  (44%, 64%) | 12.5  (9.6, 16.3) | 3  (2.4, 3.8) | 44%  (34%, 54%) |
| Primary (n=90) | 4.4  (3.5, 5.5) | 13.9  (10, 19.1) | 3.2  (2.4, 4.2) | 42%  (33%, 53%) | 10.9  (8.3, 14.2) | 2.5  (2, 3.1) | 34%  (25%, 45%) |
| No Intervention (n=18) | 4.7  (2.3, 9.4) | 5.7  (2.8, 11.7) | 1.2  (0.8, 1.8) | 6%  (1%, 26%) | 5.8  (2.9, 11.5) | 1.3  (0.8, 1.8) | 11%  (3%, 33%) |
| *p* value (Boost *vs* Primary) | 0.74 | 0.27 | 0.15 | 0.12 | 0.46 | 0.25 | 0.18 |
| Proportion difference (95% CI) |  |  |  | 12%  (-3%, 26%)^d^ |  |  | 10%  (-4%, 24%)^e^ |
|  | | | | | | | |
| ***V. cholerae O139***  ***≥ 15 years old*** | **Day 0 (Baseline)** | **Day 14**  **(Dose 1)** | | | **Day 28**  **(Dose 2)** | | |
|  | GMT^a^  (95% CI) | GMT^a^  (95% CI) | GMF rise^b^ (95% CI) | Serocon-version^c^  (95% CI) | GMT^a^  (95% CI) | GMF rise^b^  (95% CI) | Serocon-version^c^  (95% CI) |
| Boosting (n=91) | 5.3  ( 4.1, 6.9) | 9.8  (7.3, 13.4) | 1.9  (1.5, 2.3) | 23%  (16%, 33%) | 9.2  (6.8, 12.3) | 1.7  (1.4, 2.1) | 20%  (13%, 29%) |
| Primary (n=96) | 7.3  (5.6, 9.5) | 15.5  (11.5, 20.9) | 2.1  (1.7, 2.7) | 34%  (26%, 44%) | 16  (12.3, 20.6) | 2.2  (1.8, 2.7) | 33%  (25%, 43%) |
| No Intervention (n=16) | 5.9  (2.9, 11.9) | 5.2  (2.8, 9.5) | 0.9  (0.8, 1) | 0% | 6.2  (3.2, 11.8) | 1.1  (0.8, 1.5) | 6%  (1%, 28%) |
| *p* value (Boost *vs* Primary) | 0.09 | 0.03 | 0.38 | 0.09 | 0.01 | 0.10 | 0.04 |
| Proportion difference (95% CI) |  |  |  | -11%  (-24%, 1%)^d^ |  |  | -11%  (-24%, 1%)^e^ |

^a^Geometric mean reciprocal titers.

^b^Geometric mean-fold rise from baseline to 14 days post first vaccine dose or from baseline to 14 days post second vaccine dose.

^c^Percent of subjects with ≥ 4 fold rise in titers from baseline to 14 days post first vaccine dose or from baseline to 14 days post second vaccine dose. 95% confidence intervals derived using Wilson Score method.

^d^Difference seroconversion rates (95% CI) after single dose are calculated by subtracting those following primary series from those following booster dose

^e^Difference seroconversion rates (95% CI) after two doses are calculated by subtracting those following primary series from those following booster doses
